# 459. Characteristics and Presentations of Hospitalized Children due to three predominate COVID-19 variants within a healthcare network

**DOI:** 10.1093/ofid/ofad500.529

**Published:** 2023-11-27

**Authors:** Sejal M Bhavsar, Katharine N Clouser, Hailey Connolly, Jasmine Gadhavi, Ranbir Kaur, Tara Lozy, Srividya Naganathan, Margarette Pierre, Mariawy Riollano Cruz, Pooja Shah, Anita Siu, Carly Swenson, Cathleen M Ballance

**Affiliations:** Hackensack University Medical Center, Hackensack, New Jersey; Hackensack University Medical Center, Hackensack, New Jersey; Hackensack University Medical Center, Hackensack, New Jersey; Hackensack University Medical Center, Hackensack, New Jersey; HMH-JFK University Medical Center, Edison, New Jersey; Hackensack Meridian Health, Hackensack, New Jersey; JSUMC, Neptune, New Jersey; JFK university medical center, west orange, New Jersey; Hackensack Meridian School of Medicine, Neptune, New Jersey; Rutgers, The State University of NJ/Hackensack University Medical Center, Hoboken, New Jersey; Rutgers University and K. Hovnanian Children's Hospital, Piscataway, New Jersey; Hackensack Meridian School of Medicine, Neptune, New Jersey; Hackensack Meridian Health, Hackensack, New Jersey

## Abstract

**Background:**

Variants of SARS-CoV-2 caused three major waves of infections in NJ. Children and young adults were hospitalized with varying symptoms and severity of illness depending on the variant. The objectives of this study are to describe differences in the demographic and clinical characteristics, severity of illness and outcomes in pediatric patients during the different SARS-CoV-2 waves in our hospital network.

**Methods:**

We conducted a retrospective study of pediatric patients admitted to one of our three network hospitals with SARS-CoV-2 infection or with post-COVID-19 inflammatory conditions such as Multisystem Inflammatory Syndrome in Children (MIS-C) during the different waves of infection. Patients who were asymptomatic but incidentally tested positive for SARS-CoV-2 and were hospitalized for unrelated indications were excluded. We defined the timelines as follows: Wave 1 occurred between March 1, 2020 and May 31, 2020; wave 2 occurred between November 1, 2020-January 31, 2021; and wave 3 occurred between December 1, 2021-February 28, 2022. Demographic characteristics, clinical features, and outcomes were recorded and compared with respect to the different variants.

**Results:**

Of 351 total patients included in this study, 74 were admitted during wave 1, 94 during wave 2, and 181 during wave 3. The median age of patients decreased from wave 1 (11.5 years) to wave 3 (3 years) (*p*=0.0034). 87.7% of the patients were unvaccinated. The overall incidence of admissions related to COVID-19 pneumonia decreased in wave 3 compared to wave 1 and 2. COVID-19 bronchiolitis or croup admissions occurred mostly in wave 3. No significant difference in intensive care admissions was observed in any wave. Length of stay decreased across the waves (*p*< 0.0001). Treatments required did not vary between the waves except for a decrease in antibiotic use with each subsequent wave (*p*< 0.0001).

**Conclusion:**

The impact of COVID-19 on the pediatric population differs from the adult population, and the overall numbers of hospitalized children has mirrored the peak in cases observed during each infection wave. Our study illustrates the changes in clinical presentation and severity observed with the different coronavirus variants.

**Disclosures:**

**Tara Lozy, MA in Data Science**, Eli Lilly & Company: Stocks/Bonds

